# Perineuronal Nets in Spinal Motoneurones: Chondroitin Sulphate Proteoglycan around Alpha Motoneurones

**DOI:** 10.3390/ijms19041172

**Published:** 2018-04-12

**Authors:** Sian F. Irvine, Jessica C. F. Kwok

**Affiliations:** 1 School of Biomedical Sciences, University of Leeds, Leeds LS2 9JT, UK; bs11sfi@leeds.ac.uk; 2 Centre of Reconstructive Neurosciences, Institute of Experimental Medicine, The Czech Academy of Sciences, Prague 4, Czech Republic

**Keywords:** perineuronal nets, spinal cord, alpha motoneurone, gamma motoneurone, chondroitin sulphate proteoglycans

## Abstract

Perineuronal nets (PNNs) are extracellular matrix structures surrounding neuronal sub-populations throughout the central nervous system, regulating plasticity. Enzymatically removing PNNs successfully enhances plasticity and thus functional recovery, particularly in spinal cord injury models. While PNNs within various brain regions are well studied, much of the composition and associated populations in the spinal cord is yet unknown. We aim to investigate the populations of PNN neurones involved in this functional motor recovery. Immunohistochemistry for choline acetyltransferase (labelling motoneurones), PNNs using *Wisteria floribunda* agglutinin (WFA) and chondroitin sulphate proteoglycans (CSPGs), including aggrecan, was performed to characterise the molecular heterogeneity of PNNs in rat spinal motoneurones (Mns). CSPG-positive PNNs surrounded ~70–80% of Mns. Using WFA, only ~60% of the CSPG-positive PNNs co-localised with WFA in the spinal Mns, while ~15–30% of Mns showed CSPG-positive but WFA-negative PNNs. Selective labelling revealed that aggrecan encircled ~90% of alpha Mns. The results indicate that (1) aggrecan labels spinal PNNs better than WFA, and (2) there are differences in PNN composition and their associated neuronal populations between the spinal cord and cortex. Insights into the role of PNNs and their molecular heterogeneity in the spinal motor pools could aid in designing targeted strategies to enhance functional recovery post-injury.

## 1. Introduction

Perineuronal nets (PNNs) are dense specialised extracellular matrix (ECM) structures that surround neuronal sub-populations throughout the central nervous system (CNS). First described by Golgi as reticular structures in the late 1800s [1], PNNs have since been implicated in pathologies of various neurological disorders, including Alzheimer’s disease, epilepsy and schizophrenia [2,3,4,5], as well as in traumatic CNS injuries [6], particularly spinal cord injury (SCI) models [7,8]. A key role of PNNs is their involvement in the termination of developmental plasticity, where they form an interdigitating mesh with mature somatic and dendritic contacts to confer synaptic stabilisation [9,10,11].

PNNs are composed of a compact arrangement of a variety of neural ECM proteoglycans and proteins [12,13]. These components primarily consist of chondroitin sulphate proteoglycans (CSPGs) including the hyaluronan (HA) binding CSPGs called lecticans, bound upon a long HA backbone and stabilised by the HA and proteoglycan link proteins (HAPLNs) and tenascin-R [14]. Upon this basic PNN structure, the binding of other CSPGs (such as phosphacan) are thought to provide much of the heterogeneity of PNNs [15]. CSPGs are composed of chondroitin sulphate glycosaminoglycan (CS-GAG) chains attached to a core protein that differentiates the various CSPGs from one another [15]. CS-GAGs confer a further vast degree of heterogeneity through variation of expression, chain length and sulphation patterns, even to the same core protein [16,17].

Although many studies have investigated the molecular heterogeneity of PNNs in distinct neuronal populations in regions of the brain [18,19,20,21], much of the composition and associated populations in the spinal cord is relatively unknown. Similar to the brain [22], in the spinal cord many of the known cells enwrapped by PNNs are fast-spiking inhibitory parvalbumin (PV)-positive interneurones (approximately half) [23,24], and have also been associated with calbindin-positive Renshaw cells [22]. However, in contrast to the brain, reports suggest that PNNs in the spinal cord also surround cells with large neuronal cell bodies, particularly within the ventral horn likely representing motoneurones (Mns) [22,25,26,27]. Mns are a heterogeneous population of neurones with the main subclasses, alpha and gamma Mns, innervating contractile extrafusal fibres and proprioceptive intrafusal fibres within the motor unit, respectively [28].

Enzymatic removal of PNNs using chondroitinase ABC (ChABC) after CNS injury has been shown in multiple models, predominately SCI, to reopen a window of plasticity to promote improvements in motor functions [7,8]. Regeneration of descending tracts can contribute to this functional recovery [29,30]; however, the extent and mechanism of changes in local spinal circuitry attributing to this recovery remains unclear. Additionally, studies also implicate exercise and rehabilitative training to activity-dependant modulation of PNNs in the ventral motor pools [31,32], suggesting a relationship between PNNs and Mns that is important for normal motor functions.

This study therefore aims to investigate the normal expression and molecular composition of PNNs in the spinal motor pools; the population of PNN-associated neurones in the spinal cord likely to be involved in functional motor recovery after SCI, and to identify the best PNN marker for this population. Immunohistochemical staining was performed using antibodies against choline acetyltransferase (ChAT), a marker of spinal Mns [33], alongside labelling for primary PNN components, including various CSPGs and the acclaimed “universal” PNN marker *Wisteria floribunda* agglutinin (WFA), to elucidate the composition of PNNs associated with spinal motor circuitry. Selective staining for the primary functional Mn subclasses was combined with PNN labelling to categorise PNN expression within the motor pool. It was found that distinct populations of Mns were surrounded by PNNs labelled by various CSPGs yet lacking WFA, indicating a difference between the composition of PNNs and associated neuronal cell types in the brain and spinal cord. PNNs were found to surround the majority of alpha Mns, suggesting that these are the main populations affected by ChABC-mediated recovery after SCI.

## 2. Results

We aim to determine the molecular heterogeneity of PNNs in the spinal cord, with a particular focus to the Mns in the ventral horn. Spinal cord sections from three different spinal levels, cervical, thoracic and lumbar, were used to compare the spatial differences of PNNs. Alongside ChAT staining, we also stained for WFA, a common PNN marker [6,7,10,34], and for CSPGs including aggrecan (ACAN), brevican (BCAN), neurocan (NCAN), versican (VCAN) and phosphacan (PTPRZ).

### 2.1. WFA-Positive PNNs Only Partially Overlap with Other CSPGs in the Ventral Motor Pools

#### 2.1.1. ACAN

**Figure 1:**
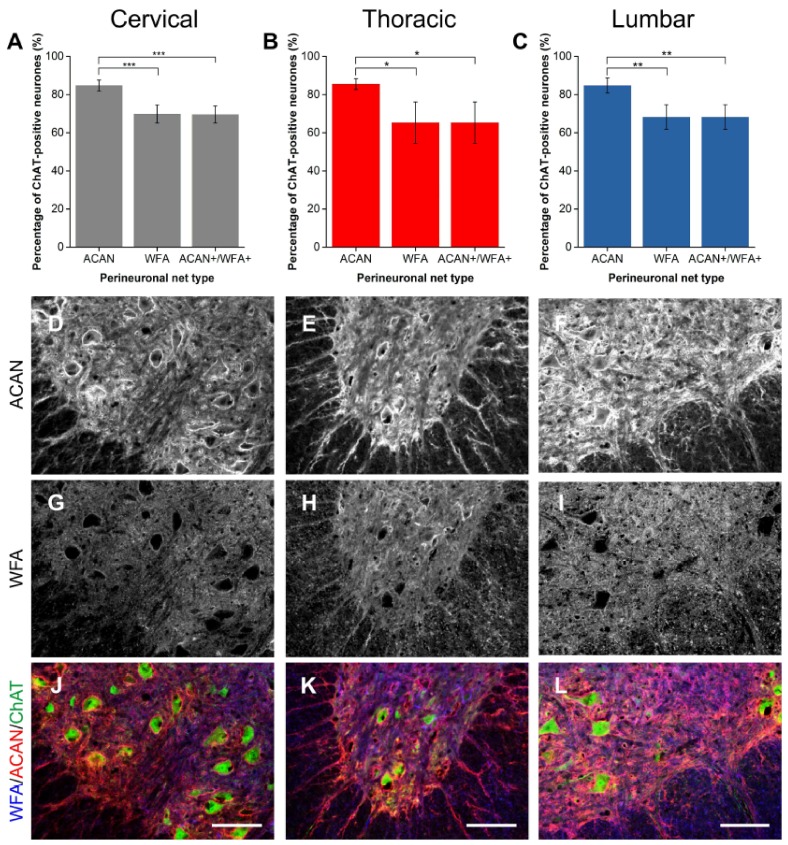
Comparison of perineuronal nets (PNNs) in the spinal ventral motor pools labelled by *Wisteria floribunda* agglutinin (WFA) and aggrecan (ACAN). (**A**–**C**) Bar graphs showing percentage of ChAT-positive motoneurones (Mns) in the ventral motor pools surrounded by ACAN-positive and WFA-positive PNNs and their co-localisation (ACAN+/WFA+) in cervical (**A**), thoracic (**B**) and lumbar (**C**) rat spinal cord. Error bars ± SD; *n* = 4. Statistics one-way ANOVA; significance levels: * *p* < 0.05, ** *p* < 0.01, *** *p* < 0.001. Confocal images showing ACAN-positive (**D**–**F**) and WFA-positive (**G**–**I**) PNNs surrounding ChAT-positive Mns (**J**–**L**) in the cervical, thoracic and lumbar spinal cord, respectively. Scale bars, 100 µm.

ACAN is a CSPG in the lectican family and is widely considered to be a major component in PNNs [2,35,36]. Immunohistochemical staining of ACAN core protein illustrated clear expression of PNNs surrounding ventral Mns labelled with ChAT (Figure 1J–L). ACAN-positive PNNs surrounded approximately 85% of ChAT-positive Mns in all levels of the spinal cord investigated (Figure 1A–C). In comparison, WFA-positive PNNs enwrapped significantly fewer Mns (approximately 68% of the ChAT-positive Mns) than ACAN-positive PNNs (cervical *p* < 0.001, thoracic *p* < 0.05 and lumbar *p* < 0.01; *n* = 4), illustrating that WFA does not label all PNNs in the ventral motor pools. Compounding this, the total number of ACAN-positive PNNs surrounding Mns was significantly greater than the number of ACAN+/WFA+ PNNs (cervical *p* < 0.001, thoracic *p* < 0.05, lumbar *p* < 0.01; *n* = 4). ACAN and WFA PNN populations appeared to overlap (Figure 1D–I). Further breakdown of PNN type revealed that, at each level, all PNNs that are positive for WFA co-localised with ACAN (n.s.; *p* = 1). No investigated PNNs were WFA-positive and ACAN-negative. The results demonstrate that ACAN labels a larger population of PNN-positive Mns, and suggest that it is a better marker for PNN in the spinal cord.

#### 2.1.2. BCAN

**Figure 2:**
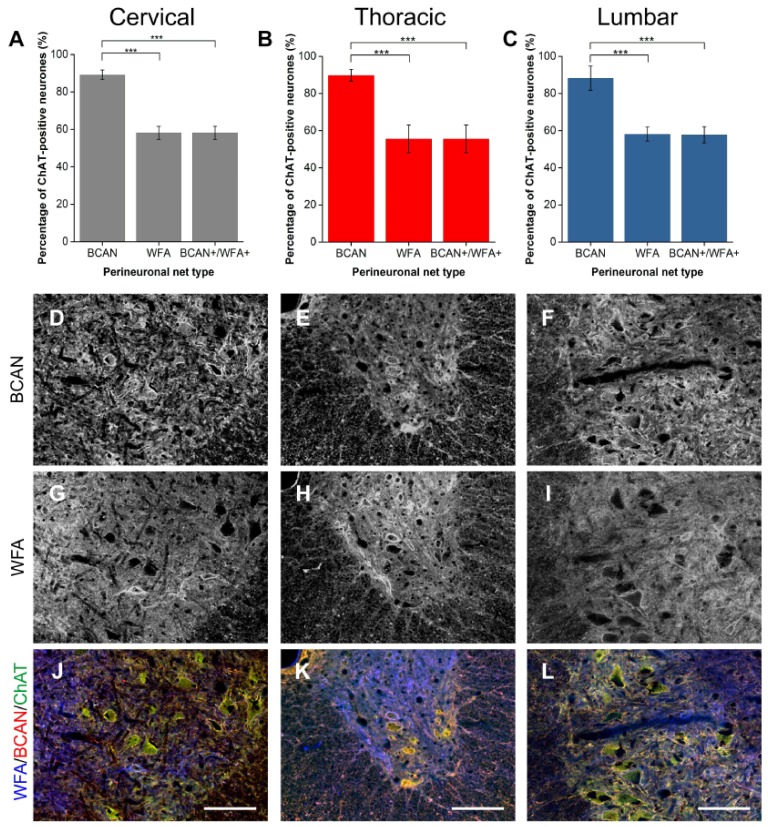
Comparison of perineuronal nets (PNNs) in the spinal ventral motor pools labelled by *Wisteria floribunda* agglutinin (WFA) and brevican (BCAN). (**A**–**C**) Bar graphs showing percentage of ChAT-positive motoneurones (Mns) in the ventral motor pools surrounded by BCAN-positive and WFA-positive PNNs and their co-localisation (BCAN+/WFA+) in cervical (**A**), thoracic (**B**) and lumbar (**C**) rat spinal cord. Error bars ± SD; *n* = 5. Statistics one-way ANOVA; significance levels: *** *p* < 0.001. Confocal images showing BCAN-positive (**D**–**F**) and WFA-positive (**G**–**I**) PNNs surrounding ChAT-positive Mns (**J**–**L**) in the cervical, thoracic and lumbar spinal cord, respectively. Scale bars, 100 µm.

BCAN is a lectican CSPG found specifically in the CNS with growing evidence of its importance in regulating the plastic properties of PNNs [37]. Co-staining with ChAT-positive neurones in the ventral horn revealed a high degree of localisation, with approximately 88% of Mns encircled by BCAN-positive PNNs (Figure 2A–C). Similar to ACAN, WFA-positive PNNs appeared to denote some but not all of the BCAN-positive PNN-ensheathed Mns, labelling approximately 30% fewer Mns than BCAN (all levels *p* < 0.001; *n* = 5). BCAN+/WFA+ PNNs in the motor pools appeared to represent a proportion that is significantly less that the total BCAN-positive PNN population (all levels *p* < 0.001; *n* = 5). Additional categorisation again revealed that all WFA-positive PNNs in the motor pool co-localised with BCAN-positive PNNs.

#### 2.1.3. NCAN

**Figure 3:**
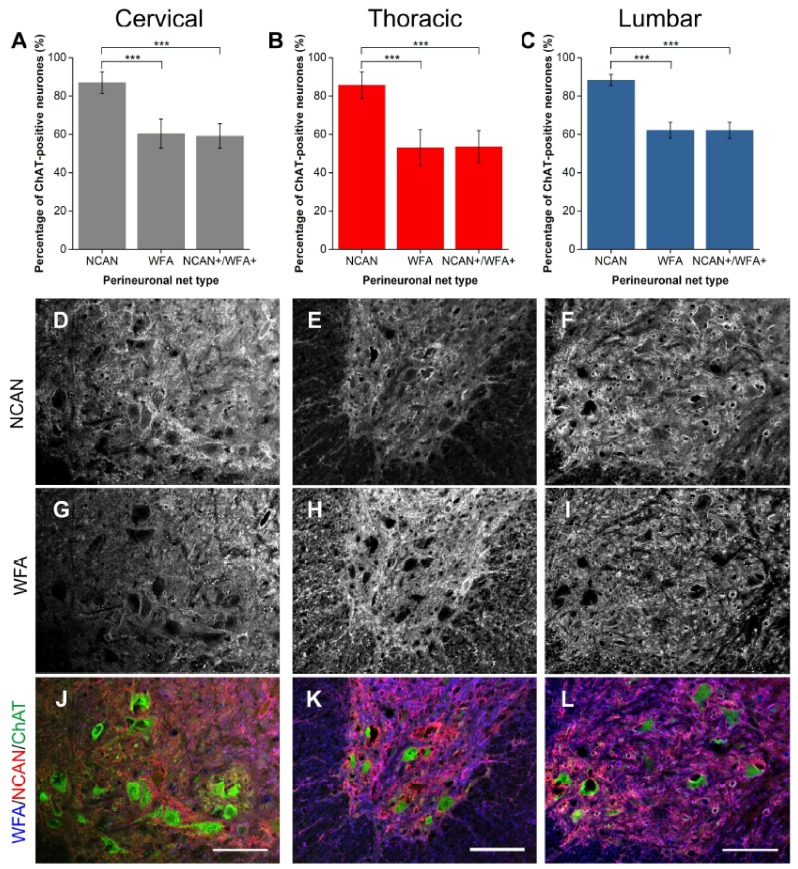
Comparison of perineuronal nets (PNNs) in the spinal ventral motor pools labelled by *Wisteria floribunda* agglutinin (WFA) and neurocan (NCAN). (**A**–**C**) Bar graphs showing percentage of ChAT-positive motoneurones (Mns) in the ventral motor pools surrounded by NCAN-positive and WFA-positive PNNs and their co-localisation (NCAN+WFA+) in cervical (**A**), thoracic (**B**) and lumbar (**C**) rat spinal cord. Error bars ± SD; *n* = 5. Statistics: one-way ANOVA; significance levels: *** *p* < 0.001. Confocal images showing NCAN-positive (**D**–**F**) and WFA-positive (**G**–**I**) PNNs surrounding ChAT-positive Mns (**J**–**L**) in the cervical, thoracic and lumbar spinal cord, respectively. Scale bars, 100 µm.

NCAN is a nervous system-specific lectican, like BCAN, known to be present in PNNs in the spinal cord [18,22,38]. In the ventral horn, NCAN staining revealed PNNs encircling approximately 87% of Mns (Figure 3A–C). Echoing the trend with ACAN and BCAN, WFA-positive PNNs enveloped 28% fewer Mns than NCAN (all levels *p* < 0.001; *n* = 5). Significantly, only approximately two-thirds of these NCAN-positive PNNs co-localised with WFA (all levels *p* < 0.001; *n* = 5). No WFA-positive PNNs lacking NCAN co-staining were observed, signifying that all WFA co-localised with NCAN.

#### 2.1.4. VCAN

**Figure 4:**
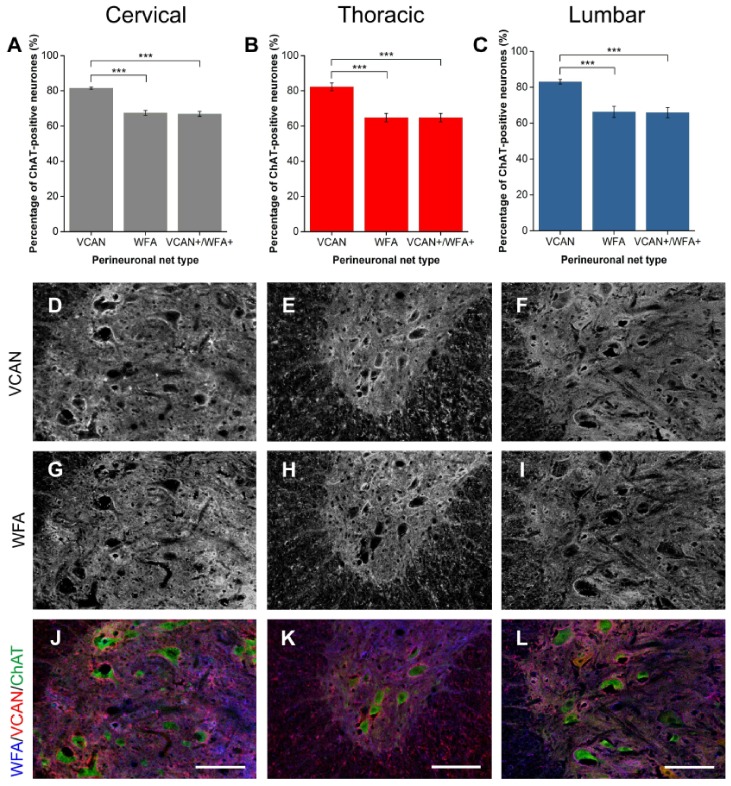
Comparison of perineuronal nets (PNNs) in the spinal ventral motor pools labelled by *Wisteria floribunda* agglutinin (WFA) and versican (VCAN). (A-C) Bar graphs showing percentage of ChAT-positive motoneurones (Mns) in the ventral motor pools surrounded by VCAN-positive and WFA-positive PNNs and their co-localisation (VCAN+/WFA+) in cervical (**A**), thoracic (**B**) and lumbar (**C**) rat spinal cord. Error bars ± SD, *n* = 4. Statistics: one-way ANOVA; significance levels: * *p* < 0.05, ** *p* < 0.01, *** *p* < 0.001. Confocal images showing VCAN-positive (**D**–**F**) and WFA-positive (**G**–**I**) PNNs surrounding ChAT-positive Mns (**J**–**L**) in the cervical, thoracic and lumbar spinal cord, respectively. Scale bars, 100 µm.

VCAN staining revealed intense diffuse ECM expression in both the white and gray matter of the spinal cord due to its expression in the nodes of Ranvier [18,39,40]. VCAN did not show strong PNN staining in laminae other than the ventral horn. In the ventral horn, VCAN-positive PNNs surrounded approximately 82% of Mns at all spinal levels (Figure 4A–C). WFA and VCAN populations of PNNs showed a clear overlap at all spinal levels (Figure 4D–L). However, all WFA-positive PNNs co-localised with VCAN with a significant population of VCAN-positive PNNs WFA-negative (all levels *p* < 0.001; *n* = 4). 

#### 2.1.5. PTPRZ

**Figure 5:**
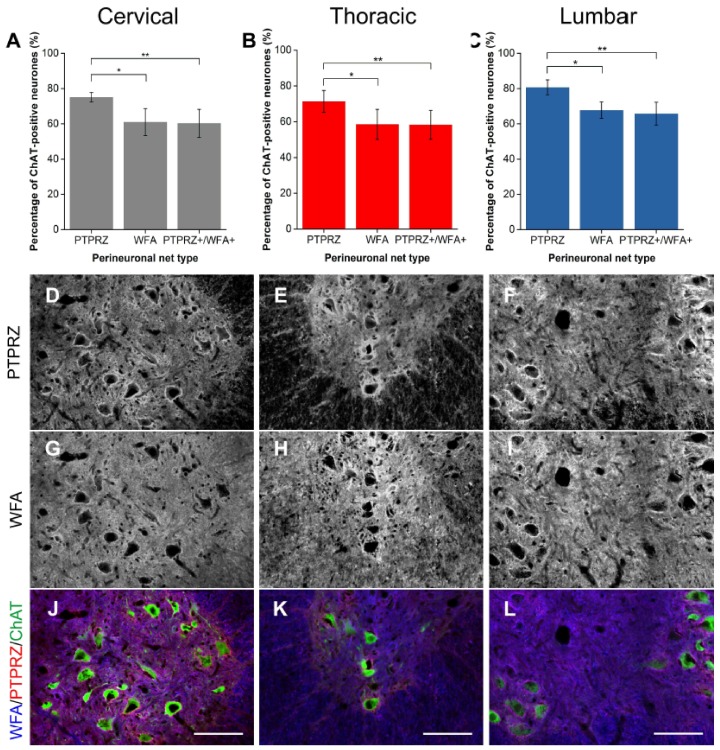
Comparison of perineuronal nets (PNNs) in the spinal ventral motor pools labelled by *Wisteria floribunda* agglutinin (WFA) and phosphacan (PTPRZ). (**A**–**C**) Bar graphs showing percentage of ChAT-positive motoneurones (Mns) in the ventral motor pools surrounded by PTPRZ-positive and WFA-positive PNNs and their co-localisation (PTPRZ+/WFA+) in cervical (**A**), thoracic (**B**) and lumbar (**C**) rat spinal cord. Error bars ± SD; *n* = 4. Statistics: one-way ANOVA; significance levels: * *p* < 0.05, ** *p* < 0.01. Confocal images showing PTPRZ-positive (**D**–**F**) and WFA-positive (**G**–**I**) PNNs surrounding ChAT-positive Mns (**J**–**L**) in the cervical, thoracic and lumbar spinal cord, respectively. Scale bars, 100 µm.

Phosphacan or PTPRZ is a non-HA binding CSPG that represents the extracellular domain of protein tyrosine phosphatase receptor zeta (PTPRZ) modified by glial cells [41,42] and has been found to be present in WFA-positive PNNs in the cerebral cortex [18,22,43]. Immunohistochemistry showed that PTPRZ is also found in PNNs in the ventral motor pool, surrounding approximately 76% of Mns in all levels of the cord studied (Figure 5A–C). However, in all levels of the spinal cord investigated, PTPRZ-positive PNNssurrounded 15% more Mns than WFA (all levels *p* < 0.01; *n* = 4), reiterating the trend shown by the lecticans above. Approximately 82% of PTPRZ-positive PNNs were also labelled by WFA, representing a significantly lower proportion of the total observed PTPRZ-positive PNNs in the motor pool (all level *p* < 0.05; *n* = 4).

### 2.2. Distinct Populations of CSPG-Positive yet WFA-Negative PNNs in the Motor Pools

**Figure 6:**
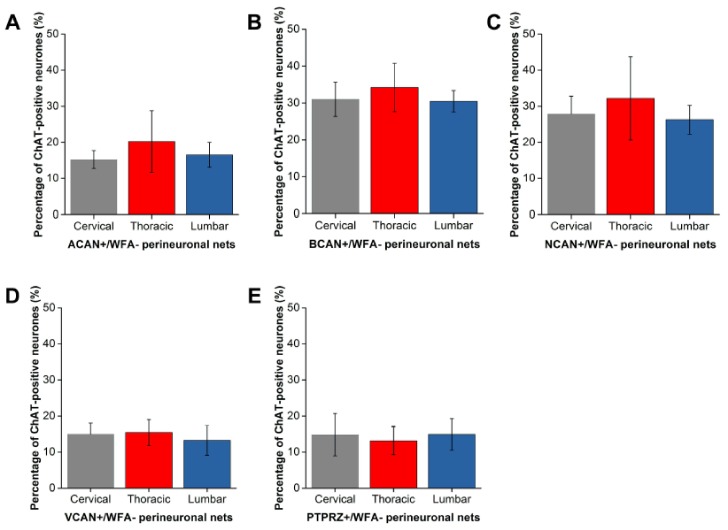
A proportion of perineuronal nets (PNNs) in the spinal motor pools were negative for *Wisteria floribunda* agglutinin (WFA). Percentage of ChAT-positive motoneurones in the ventral motor pools in the cervical, thoracic and lumbar spinal cord surrounded by CSPG-positive, WFA-negative PNNs. (**A**) Aggrecan (ACAN, *n* = 4); (**B**) brevican (BCAN, *n* = 5); (**C**) neurocan (NCAN, *n* = 5); (**D**) versican (VCAN, *n* = 4); and (**E**) phosphacan (PTPRZ, *n* = 4). Error bars ± SD. Statistics one-way ANOVA; n.s.

For each CSPG investigated, a significant percentage of Mns were surrounded by PNNs that were CSPG-positive yet WFA-negative (all levels, all CSPGs *p* < 0.001). The percentage of Mns with WFA-negative PNNs varied with CSPG investigated (Figure 6). While ACAN+/WFA−, VCAN+/WFA− and PTPRZ+/WFA− PNNs encircled roughly 15% of Mns (Figure 6A,D,E), a higher percentage of Mns (approximately 30%) appeared to be surrounded by BCAN+/WFA− and NCAN+/WFA− PNNs (Figure 6B,C). Overall, the results suggest that in the ventral motor pools, WFA does not denote all PNNs, and instead distinct populations of Mns with CSPG-positive, WFA-negative PNNs exist.

### 2.3. Alpha Mns Are Preferentially Surrounded by PNNs

**Figure 7:**
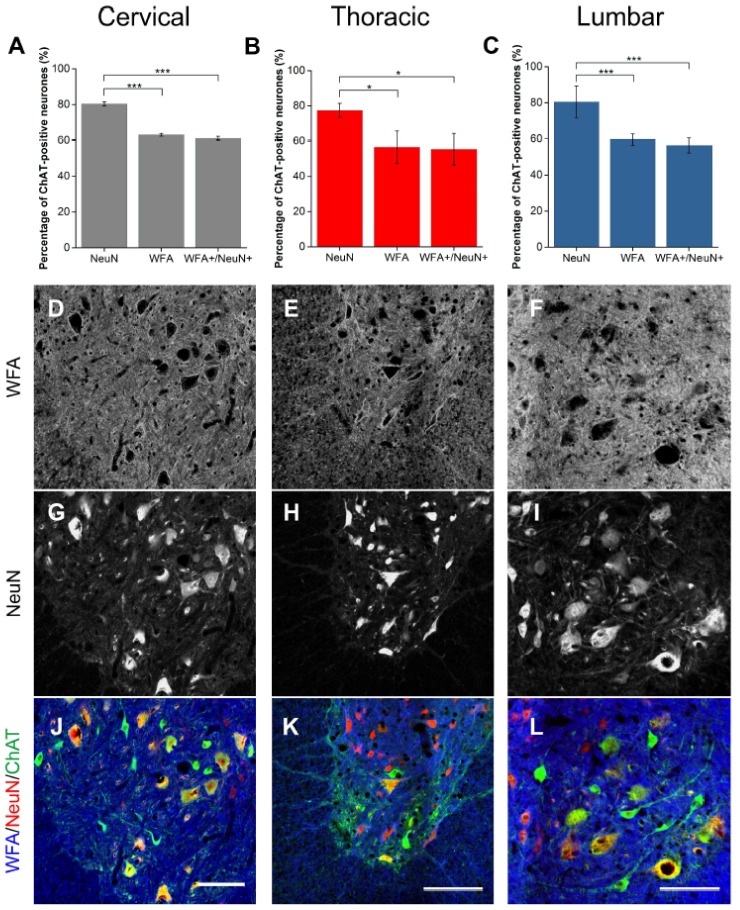
*Wisteria floribunda* agglutinin (WFA)-positive PNNs surrounded some but not all alpha motoneurones (Mns). (**A**–**C**) Bar graphs showing percentage of ChAT-positive Mns in the ventral motor pools surrounded by NeuN, WFA-positive PNNs and their co-localisation (WFA+/NeuN+) in cervical (**A**), thoracic (**B**) and lumbar (**C**) rat spinal cord. NeuN and ChAT co-localisation denotes alpha Mns. Error bars ± SD; *n* = 3. Statistics: one-way ANOVA; significance levels: * *p* < 0.05, *** *p* < 0.001. Confocal images showing WFA-positive PNNs (**D**–**F**) surrounding NeuN-positive (**G**–**I**) PNNs and ChAT-positive Mns (**J**–**L**) in the cervical, thoracic and lumbar spinal cord, respectively. Scale bars, 100 µm.

**Figure 8:**
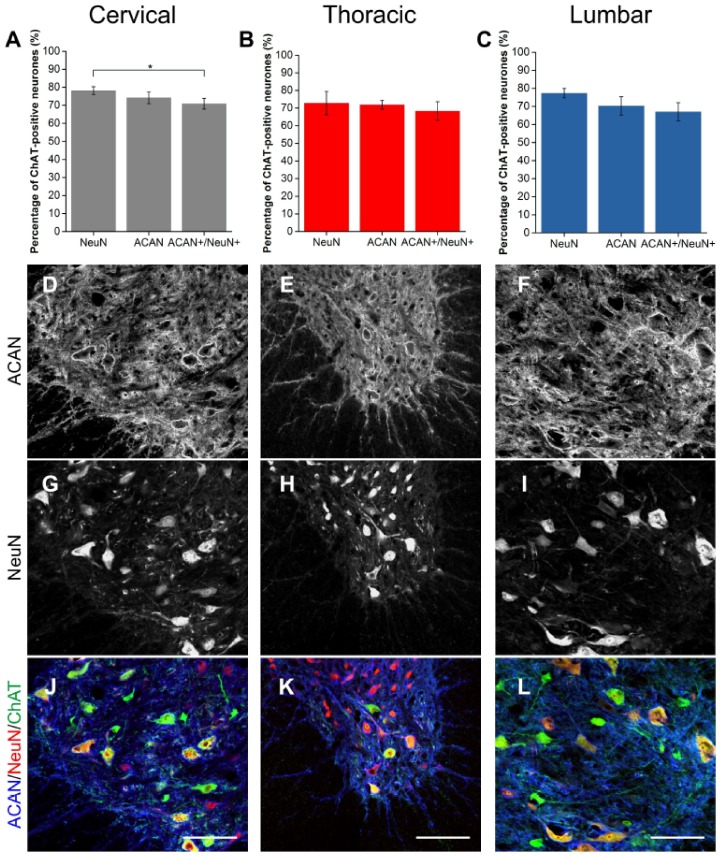
Aggrecan (ACAN)-positive PNNs surrounded most alpha motoneurones (Mns). (**A**–**C**) Bar graphs showing percentage of ChAT-positive Mns in the ventral motor pools surrounded by NeuN, ACAN-positive PNNs and their co-localisation (ACAN+/NeuN+) in cervical (**A**), thoracic (**B**) and lumbar (**C**) rat spinal cord. NeuN and ChAT co-localisation denotes alpha Mns. Error bars ± SD; *n* = 3. Statistics: one-way ANOVA; significance levels: * *p* < 0.05. Confocal images showing ACAN-positive PNNs (**D**–**F**) surrounding NeuN-positive (**G**–**I**) PNNs and ChAT-positive Mns (**J**–**L**) in the cervical, thoracic and lumbar spinal cord, respectively. Scale bars, 100 µm.

Using NeuN and ChAT co-labelling, Mns in the spinal ventral motor pools were selectively labelled as either alpha (NeuN-positive) or gamma (NeuN-negative) [31,44]. It was observed that approximately 70–80% of ChAT-positive Mns were NeuN-positive (Figure 7 and Figure 8), signifying thealpha Mn population. Firstly, as the universal marker for PNNs, WFA was used to determine the number of PNNs surrounding each Mn subtype. Similarly to the results above, WFA-positive PNNs surrounded approximately 60% of all Mns with approximately 98% of these PNNs surrounding NeuN-positive Mns (alphas; Figure 7A–C). In other words, a significant proportion of alpha Mns (~72%) were associated with WFA-positive PNNs (cervical and lumbar *p* < 0.001, thoracic *p* < 0.05; *n* = 3). As previous findings illustrated that in the ventral motor pools, WFA did not label all Mns, ACAN was also used to identify PNNs around Mn subtype. Again, most PNNs (95%) surrounded alpha Mns (Figure 8A–C). ACAN-positive PNNs encircled roughly 90% of alpha Mns, suggesting that PNN-positive Mns and alpha Mns are the same population.

## 3. Discussion

As removal of PNNs in the spinal cord after injury enhances motor recovery, we looked to investigate the expression of PNNs and their heterogeneity in spinal Mns; the final order neurones for the control of voluntary movement. This is the first article to systemically and quantitatively compare the differences of CSPG- or WFA-positive PNN Mns in the ventral motor pools. Mns were identified using an antibody against ChAT to label cholinergic neurones alongside markers for PNN components and the acclaimed universal PNN marker WFA in comparison. We demonstrated that a high proportion of Mns in the ventral spinal cord were surrounded by PNNs, particularly alpha Mns. Unexpectedly, the universal marker for PNNs, WFA, did not label all of the PNNs with distinct populations of Mns surrounded by CSPG-positive yet WFA-negative PNNs. This suggests that, in contrast to the brain, WFA does not label the majority of PNN neurones in the ventral spinal cord and that studies using WFA in the spinal cord may be underestimating the number of PNNs.

### 3.1. PNNs in the Spinal Ventral Motor Pools

Previous studies have described ventral Mns as the most conspicuous neuronal population in the spinal cord to be surrounded by PNNs and this appears to be conserved across mammalian species [25,31,32,45,46]. Despite this, few studies have actually investigated the proportion of PNNs in the ventral motor pools and those that do use varying markers to determine this. Comparable to our own methods, using ChAT as a specific Mn marker, a similar proportion of ventral Mns was observed to be surrounded by PNNs to that found in our study (~80%) has been reported in non-human primates (75%), using WFA [45], and in human (71%) spinal cord, using ACAN [46]. In rats, however, this distribution has been investigated with the general neuronal marker (NeuN) using size and ventral location to identify Mns alongside WFA lectin staining to characterise PNN expression, resulting in estimates of only 30% of Mns associated with PNNs [25]. This is likely to underestimate for two reasons: (1) without a Mn-specific neuronal marker small sized Mns, including NeuN-negative gamma Mns [44,47], would have been absent from these counts, and (2) WFA does not appear to label all PNNs in the rat spinal cord. Indeed, our findings suggest that PNNs are present in almost 80% of the ChAT-positive Mns.

Although others have implicated that PNNs only surround large cell-bodied Mns in the motor pools, i.e., alpha Mns and not gamma Mns [25,31], we are the first to systematically categorise the proportion of specific Mn-subtypes associated with PNNs at different levels of the spinal cord. Despite contributing to the same goal of voluntary muscle control, alpha and gamma Mns represent distinct populations of Mns within the ventral motor pools, differing in both electrical and molecular properties [28,44,48,49]. These differences also include the innervation of different muscle targets, with alpha Mns responsible for force generation though contraction of extrafusal fibres whereas gamma Mns innervate the intrafusal fibres regulating muscle spindle sensitivity. The high proportion of enveloped alpha Mns revealed likely reflects the importance of the role of PNNs in providing synaptic stabilisation of inputs from the specific spinal circuitry and consequent contractile innervation of key muscle groups. After SCI, the stabilisation of synaptic plasticity conferred by PNNs instead becomes another mechanism inhibiting regenerative attempts and compensatory rearrangements of spared fibres. We suggest that ChABC-mediated removal of PNNs in SCI models is therefore able to induce a high degree of enhanced plasticity of synaptic connections to the abovementioned populations of alpha Mns contributing to the observed improvement of most functional motor recovery studies.

### 3.2. Differences in PNNs between the Brain and Spinal Cord

It is generally assumed that PNNs in the brain and the spinal cord are the same. While in the brain, WFA does not always co-localise with ACAN as previously discussed, other CSPG-positive PNNs always co-localise with WFA. Here, we demonstrate differences in the composition of PNNs between the brain and spinal cord, where ACAN and other CSPGs denote subclasses of Mns in the spinal cord lacking WFA. This study recommends that future staining for PNNs associated with the spinal motor pools, particularly SCI studies utilising therapies that modify PNNs such as ChABC, should seek alternatives to WFA to avoid underestimating total PNN number.

Additionally, brain PNNs are well known to target small fast-spiking inhibitory interneurones playing a modulatory role in the brain [50]. In sharp contrast, the associated neuronal populations studied here are large cell bodied neurones acting as the primary endpoint of neural control of the somatic motor system. Other neuronal cell types such as calbindin-positive Renshaw cells in the ventral spinal cord are surrounded by PNNs [22], further implicating the role of PNNs in stabilisation of connections within the spinal motor circuitry. In a recent systematic review of the CNS, motor regions, including the cerebellum and spinal cord, were more likely to have a higher proportion of neurones surrounded by PNNs than sensory structures [45]. It is possible that PNNs may have different roles in different parts of the nervous system or with different neuronal populations.

### 3.3. Composition of PNNs in the Spinal Motor Pools

Staining with the lectin WFA and antibodies for various CSPG core proteins revealed two distinct types of distributions throughout the grey matter: diffuse extracellular staining and a bright ‘halo’ of pericellular expression identifying the PNNs. The overall distributions of immunoreactivities for the CSPGs investigated and ChAT were generally similar to previous descriptions [18,22,25,33]. We showed that all of the CSPGs investigated were present in PNNs surrounding spinal Mns. These were also found to be present to varying degrees, indicating heterogeneity of PNNs in the motor pools.

ACAN in particular has been previously reported to be present in PNNs surrounding Mns [22,51,52], as well as BCAN, NCAN, VCAN and PTPRZ. It is estimated that VCAN begins to appear in PNNs around the Mns from postnatal day 8 [53]. Studies in the brain and spinal cord show that ACAN is present in all PNNs and generally co-localises with WFA expression [12,18,25]. However, consistent with all CSPGs investigated, WFA does not appear to show all PNN-associated neurones in the ventral motor pools. As WFA is supposed to bind to the CS-GAG sugar *N*-acetylgalactosamine (GalNAc) [50], it should bind to all CSPGs and therefore denote all PNNs. However, binding of WFA has previously been shown to be dependent on the presence of ACAN [12] and recently other studies in various regions of the brain, including the hippocampus, have reported PNNs with ACAN labelling but no WFA binding [21,54]. In the spinal cord, we observed a lack of WFA in ACAN-positive PNNs to a similar degree to that observed in the CA1 area of the hippocampus [21], appearing to denote distinct populations of Mns. As there is a vast degree of heterogeneity of CS-GAGs within CSPGs, further research is required to determine the conditions of WFA binding. It is possible that the molecular composition of CSPGs within PNNs may confer functional subclasses of Mns.

The expression of many PNN components such as ACAN, BCAN and tenascin-R show differences in expression between various brain regions [55]. In particular, BCAN is usually found at the para-nodal regions and has been shown to regulate the localisation of potassium channels and AMPA receptors [37]. The mechanism of how brevican performs these functions remains to be determined. Expression of CSPGs in PNNs across the spinal laminae has also been shown to display differential expression [22,25]. PNNs are a dynamic network of ECM components. Activity-dependant modulation has been demonstrated where the thickness of PNNs surrounding spinal Mns increases in response to exercise or rehabilitative training [31,32]. This is likely conveyed through dynamic regulation of CSPGs and/or CS-GAGs within the PNNs. There is a growing concept that the properties of the ECM have an important influence in both healthy and pathological states. Though it is beyond the scope of this study, it is hoped that further research into the heterogeneity of PNNs in CNS regions may help to unravel the functionality of these ECM components and their alterations in disease states.

### 3.4. Further Research and Conclusions

Despite the clinical relevance of PNNs targeted for CNS repair and regeneration, particularly in locomotor recovery models of SCI, the functional relationship between PNNs and the motor system is still mostly unexplored. Further research is required to look at the normal functional properties of PNNs surrounding Mns. Additionally, the molecular heterogeneity of PNNs displayed in spinal Mns may indicate a functional role. However, understanding how the varying molecular heterogeneity of PNNs affects CNS functions is a topic still in its infancy.

Though this study begins to address a research gap surrounding the properties of PNNs in the spinal cord, much characterisation remains to be done. While ChABC has been an invaluable investigative tool for understanding the role of PNNs in promoting plasticity and functional recovery after SCI, there are clinical limitations to its therapeutic use. It is hoped that insights into the properties of PNNs and their role in the spinal cord could aid the generation of alternative and non-invasive strategies for targeted PNN removal to enhance functional recovery post-injury.

## 4. Materials and Methods

### 4.1. Animals

Female Lister Hooded rats (200–250 g; *n* = 5) were obtained from Charles River Laboratories (Canterbury, UK) and were housed in groups in Central Biomedical Services (University of Leeds) in a temperature controlled environment (20 ± 1 °C), with a 12 h light/dark cycle (lights on at 07:00). Access to food and water was *ad libitum*. All procedures and experiments complied with the UK Animals (Scientific Procedures) Act 1986.

### 4.2. Tissue Preparation

Animals were given an overdose of sodium pentobarbital (Pentoject; Henry Schein; 200 mg/kg; intraperitoneal injection) to deeply anaesthetise without halting cardiac function. A transcardial perfusion [56] was then performed using sodium phosphate buffer (PB; 0.12 M sodium phosphate monobasic; 0.1 M NaOH; pH 7.4) followed by 4% paraformaldehyde (PFA; in PB; pH 7.4) for tissue fixation. The spinal cord was dissected out, post-fixed in PFA (4%; 4 °C) overnight and cryoprotected in 30% sucrose solution (30% *v*/*w* sucrose in PB; 4 °C) until tissue saturation. The appropriate cervical (C3-T1), mid-thoracic and lumbar (L1-6) spinal cord segments were removed and frozen in optimum temperature medium (OCT; Leica FSC 22 Frozen Section Media; Leica Biosystems) before storage at −80 °C until sectioning. Tissue was cut using a cryostat (Leica CM1850; Leica Biosystems) into 40 µm transverse sections. Sections were serially collected into 48-well plates containing physiological buffer solution (PBS; 0.13 M NaCl, 0.7 M sodium phosphate dibasic, 0.003 M sodium phosphate monobasic; pH 7.4) to remove the OCT before being transferred to 30% sucrose solution for storage at 4 °C.

### 4.3. Staining Procedures

Immunohistochemical techniques were used to label for cells in the spinal cord containing ChAT and the PNNs surrounding subsets of these cells labelled by biotinylated *Wisteria floribunda* agglutinin (bio-WFA) and CSPG components, including ACAN, BCAN and NCAN (Table 1). ChAT was used for Mn identification [57] whilst WFA is universally used as a marker for PNNs [10,12].

At room temperature (RT), free-floating sections were washed three times for 5 min each in Tris-buffered saline (TBS; 0.1 M tris base, 0.15 M NaCl; pH 7.4) to remove sucrose residue. Tissue was then blocked in 0.3% TBST (1× TBS solution and 0.3% *v*/*v* Triton X-100) and 3% normal donkey serum (NDS; *v*/*v*) for two hours. The sections were then transferred to co-incubate at 4 °C in blocking buffer (3% NDS in 0.3% TBST; pH 7.4) containing the following primary antibodies: anti-ChAT (goat; Millipore; 1:500; 48 h), biotin-conjugated *Wisteria floribunda* agglutinin (bio-WFA; Sigma; 1:150; 24 h) and either ACAN (rabbit; Millipore; 1:250; 24 h), BCAN (mouse; DSHB; 1:500; 24 h), NCAN (mouse; DSHB; 1:100; 24 h), VCAN (mouse; DSHB; 1:100; 24 h) or PTPRZ (mouse; DSHB; 1:80; 24 h) (Table 1).

Immunostaining was routinely carried out using tissue from different animals and differing spinal segments. The combinations carried out in this study used the formula: ChAT—Bio-WFA—CSPG marker using various antibodies from Table 1, including for the lecticans ACAN, BCAN, NCAN and VCAN. To differentiate between alpha and gamma Mns [31,44], ChAT was co-stained with anti-NeuN (mouse; Millipore; 1:500; 24 h). Antibodies requiring 24-h incubation were added and mixed well 48 h into a 72-h incubation with ChAT, using the protocols as above. To visualise each primary antibody staining, the tissue was then co-incubated with the appropriate species of fluorescent-conjugated secondary antibodies (1:500; 2 h; RT; Table 2).

### 4.4. Image Acquisition and Quantification Methods

The fluorophores used to label the spinal cord sections were visualised using a Zeiss LSM 880 (upright) confocal microscope and were used to generate tile scans of the entire spinal cord transverse section at 20× magnification (1.03 µs per pixel, averaging: 4). ChAT-positive cells and co-localisation with WFA-positive PNNs and other CSPG-positive PNNs were counted using the Cell Counter plugin (Kurt de Vos; https://imagej.nih.gov/ij/plugins/cell-counter.html) in the software FIJI [58]. Mns were identified by location within the ventral horn of ChAT-positive cellular staining. All ChAT-positive cells were individually counted and sequentially analysed for presence of PNN staining. PNNs were only counted around ChAT-positive neurones and were identified by the presence of intense staining as a bright ‘halo’ directly adjacent to the perimeter of ChAT-positive cells. Positive PNN staining was categorised into three classes: (1) only WFA-positive, (2) only positive for the appropriate CSPG stain or (3) WFA-positive and CSPG-positive. For differentiation of alpha and gamma Mns, cells co-localising both ChAT and NeuN staining where taken as alpha Mns whereas the absence of NeuN denoted gamma Mns [44,59].

### 4.5. Experimental Design and Statistical Analysis

A minimum of three sections per spinal level (cervical, thoracic or lumbar) per animal (*n* = 5) were stained and imaged, maintaining the same confocal microscopy settings per staining procedure. All counts per section were normalised by the number of ChAT-positive cells before averaging per animal. All data sets were analysed with OriginPro 2016 scientific graphing and data analysis software (OriginLab, Northampton, MA, USA), where results were statistically significant given that *p* < 0.05. To test the influence of spinal cord level on PNN expression and for differences between PNN types, results were pooled and analysed using one-way ANOVA, with Bonferroni correction for between-groups multiple comparison.

## Figures and Tables

**Table 1:** Immunohistochemical detection of extracellular matrix components and neuronal markers, including concentration (conc.) of antibody used.

Detected Component	Marker	Host	Antibody Conc.	Source	Characterisation
CSPGs					
Aggrecan (mouse ACAN core protein)	Anti-ACAN	Rabbit polyclonal IgG	500 µg/mL	Millipore #AB1031	WB^2^ (Lendvai et al., 2013 & Sutkus et al., 2014)
Brevican (BCAN; mouse cell-line derived recombinant human Brevican)	Anti-BCAN	Sheep polyclonal IgG	1 mg/mL	R&D Systems #AF4009	WB^2^ (R&D Systems data sheet)
Neurocan (NCAN; N-terminal epitope)	Anti-NCAN	Mouse monoclonal IgG	369 µg/mL	DSHB^1^ #1F6	WB^2^ (Asher et al., 2000 & Deepa et al., 2006)
Versican (VCAN; hyaluronate-binding region)	Anti-VCAN	Mouse monoclonal IgG	169 µg/mL	DSHB^1^ #12C5	WB^2^ (Asher et al., 2002 & Deepa et al., 2006)
Phosphacan (PTPRZ)	Anti- PTPRZ	Mouse monoclonal IgG	165 µg/mL	DSHB^1^ #3F8	WB^2^ (Deepa et al., 2006 & Vitellaro-Zuccarello et al., 2006)
Lectins					
*N*-acetylgalactosamine (GalNAc)	Biotinylated *Wisteria floribunda* agglutinin (WFA)	N/A	2 mg/mL	Sigma #L1766	Koppe et al., 1996
Neuronal markers					
Choline acetyltransferase (ChAT)	Anti-ChAT	Goat polyclonal IgG	-	Millipore #AB144P	-
Neuron-specific nuclear protein (NeuN)	Anti-NeuN	Mouse monoclonal IgG	1 mg/mL	Millipore #MAB377	WB^2^ (Jin et al., 2003)

^1^ DSHB, Developmental Studies Hybridoma Bank, University of Iowa, USA. ^2^ WB, Western blotting.

**Table 2:** Fluorescent-conjugated secondary antibodies (2 mg/mL) used for immuno-detection of primary antibodies.

Antibody	Host	Source
Alexa fluor 488	chicken anti-goat IgG	Invitrogen #A21467
Alexa fluor 568	donkey anti-mouse IgG	Invitrogen #A31571
Alexa fluor 568	donkey anti-rabbit IgG	Invitrogen #A10042
Alexa fluor 568	donkey anti-sheep IgG	Invitrogen #A21099
Alexa fluor 647	*Streptavidin*-conjugated	Invitrogen #S32357

## Abbreviations

ACAN	Aggrecan core protein
BCAN	Brevican core protein
ChAT	Choline acetyltransferase
ChABC	Chondroitinase ABC
CNS	Central nervous system
CS-GAG	Chondroitin sulphate glycosaminoglycan
CSPGs	Chondroitin sulphate proteoglycans
ECM	Extracellular matrix
GalNAc	*N*-acetylgalactosamine
HA	Hyaluronic acid/hyaluronan
Mn	Motoneurone
NeuN	Neuron-specific nuclear protein
NCAN	Neurocan core protein
PTPRZ	Phosphacan/protein tyrosine phosphatase receptor zeta
PNN	Perineuronal net
Mn	Motoneurone
SCI	Spinal cord injury
VCAN	Versican core protein
WFA	*Wisteria floribunda* agglutinin
